# Naturalistic Study of Depression Associated with Parkinson’s Disease in a National Public Neurological Referral Center in Mexico

**DOI:** 10.3390/brainsci12030326

**Published:** 2022-02-28

**Authors:** Reinhard Janssen-Aguilar, Patricia Rojas, Elizabeth Ruiz-Sánchez, Mayela Rodriguez-Violante, Yessica M. Alcántara-Flores, Daniel Crail-Meléndez, Amin Cervantes-Arriaga, Óscar Sánchez-Escandón, Ángel A. Ruiz-Chow

**Affiliations:** 1 Department of Psychiatry, National Institute of Neurology and Neurosurgery Manuel Velasco Suárez, Av. Insurgentes Sur No. 3877, Mexico City 14269, Mexico; rjanssen91@gmail.com (R.J.-A.); danielcrail@yahoo.com (D.C.-M.); 2 Laboratory of Neurotoxicology, National Institute of Neurology and Neurosurgery Manuel Velasco Suárez, Av. Insurgentes Sur No. 3877, Mexico City 14269, Mexico; prcastane@hotmail.com (P.R.); elizabeth.ruiz@innn.edu.mx (E.R.-S.); fioremyr@gmail.com (Y.M.A.-F.); 3 Movement Disorders Clinic, National Institute of Neurology and Neurosurgery Manuel Velasco Suárez, Av. Insurgentes Sur No. 3877, Mexico City 14269, Mexico; mrodriguez@innn.edu.mx (M.R.-V.); acervantes@innn.edu.mx (A.C.-A.); 4 Clinic of Sleep Disorders, Metropolitan Autonomous University, Mexico City 14387, Mexico; oscarse@att.net.mx; 5 Liaison Psychiatry, Medical Center ABC, Av. Carlos Graef Fernández 154, Mexico City 05300, Mexico

**Keywords:** Parkinson’s disease, non-motor symptoms, neurodegenerative disease, major depressive disorder

## Abstract

Major depressive disorder (MDD) is a major health problem in Parkinson’s disease (PD) patients. We described the clinical and sociodemographic factors of MDD among patients with PD at a national neurological referral center in Mexico. One hundred patients with PD + MDD were included in the study. All the patients were evaluated during the “ON” treatment phase of PD. Clinical scales for cognition (MMSE and MoCA) and MDD (MADRS) were applied. The mean age was 58.49 ± 11.02 years, and 57% of the sample was male. The most frequent symptom of PD was tremor (67%), and onset was more frequent on the right side (57%). Additionally, 49% of the patients with PD had moderate to severe (M/S) MDD. Selective serotonin reuptake inhibitors were the most frequent antidepressant treatment (69%). The scores of the scales were MADRS 21.33 ± 5.49, MoCA 21.06 ± 4.65, and MMSE 26.67 ± 1.20. The females had lower MMSE scores compared to the males (*p =* 0.043). The patients with M/S MDD had more rigidity at the beginning of PD (*p =* 0.005), fewer march alterations (*p =* 0.023), and a greater prevalence of left-side initial disease (*p =* 0.037). Rigidity was associated with M/S MDD (OR 3.75 *p =* 0.013). MDD was slightly more frequent in the males than in the females. The MDD symptoms and cognitive impairment were worse in the female population.

## 1. Introduction

Parkinson’s disease (PD) is a complex illness and the second most common neurodegenerative disorder, affecting 1% of the population over 60 and up to 4% over the age of 80 [1]. It is estimated that between 1 and 2% of the population in Mexico over the age of 60 suffers from PD [2], and the prevalence of the disease increases with age. The etiology of PD is currently unclear and no currently available treatment provides a cure [3]. In addition to classical motor symptoms (bradykinesia, rest tremor, or rigidity), the presence of non-motor features, such as hyposmia, sleep behavior disorder, cognitive impairment, pain, autonomic dysfunction, and psychiatric disturbances, are relevant. Psychiatric symptoms, such as major depressive disorder (MDD), anxiety, hallucination, delusion, apathy and anhedonia, impulsive and compulsive behavior, and cognitive dysfunction, appear to be present in most PD patients [4].

MDD is a major health problem in patients with PD. The predictors of MDD in PD are debatable and complex [3], although the prevalence of MDD in PD patients has been reported to be 20–35%, and the one-year incidence of minor MDD is 18%. It should also be mentioned that the prevalence and incidence of MDD in these patients vary depending on the diagnostic criteria [4]. However, MDD is not exclusive to the population over 60 years with PD and may occur in a population under 50 years of age, as in the case of early-onset and juvenile- and young-onset PD. In this population of onset before the age of 50, a prevalence of MDD up to 45.6% has been found [5]. This psychiatric disorder can manifest at any time, from the pre-motor stage to late stages of the disease [6], and generally involves apathy, anhedonia, and somatic and neurovegetative symptoms, such as fatigue, difficulty concentrating, and insomnia. Therefore, it may be challenging to identify clinical MDD in PD patients [7].

In particular, MDD appears to be one of the most important factors impairing both the subjective and objective quality of life, independent of motor deficits. It is likely that MDD in PD is multifactorial, and the triggers include motor deficits, disability, the burden on caregivers, economic strain, cognitive impairment, and the severity of the medical illness. Therefore, there is a need to study diverse associated factors, such as age, sex, disease severity, longer disease duration, a younger PD onset age, frequent falls, lower educational level, and regular use of non-aspirin bases (NSAIDs) or analgesics [3]. Then, this disease would not be underdiagnosed and undertreated in clinical practice. The aim of this study is to describe and examine the clinical and sociodemographic factors in major depressive disorder among patients with PD in the outpatient clinic of a national neurological referral center in Mexico. Evaluating MDD and identifying the risk factors for developing MDD is important for the Mexican population.

## 2. Materials and Methods

### 2.1. Participants

We carried out a cross-sectional observational study on 100 consecutive depressed PD subjects, evaluated for the first time at the Movement Disorders outpatient clinic at the National Institute of Neurology and Neurosurgery Manuel Velasco Suarez (INNNMVS) in Mexico City, Mexico. The study followed the principles of the Declaration of Helsinki and its later amendments. The protocol was approved by the ethics committee of INNNMVS (approval number 100/11). All participants signed informed consent for inclusion in the study.

Patients were recruited from 2016 to 2018. Diagnoses of PD were established by a specialist in movement disorders (according to the UK PD Brain Bank Criteria) [8], and diagnosis of MDD was made by a neuropsychiatrist (using the Montgomery–Asberg Depression Rating Scale, MADRS) [9]. Patients were excluded when they had a diagnosis of neurological diseases other than PD, had a follow-up of abnormal movements less than 1 year in the clinic, had a diagnosis of psychiatric diseases that were previously diagnosed, took antiparkinsonian medication with an antidepressant effect, or had modifications in antiparkinsonian drugs within 4 weeks of the start of antidepressant treatment.

In addition to standard assessment, a semi-structured interview was used to obtain information on the disease history (age of onset of PD, disease duration, family history of PD, symptoms at the beginning of PD, history of chronic degenerative diseases, PD treatment, previous MDD treatment, use of antidepressants at outpatient clinics, years on PD treatment, and history of psychiatric illness) and other sociodemographic data (age, gender, marital status, education level, alcohol use, caffeine use). All patients were assessed using the MDS-UPDRS (unified Parkinson´s disease rating scale) part III scale for motor symptoms (completed in the “ON” period), MADRS, mini-mental state examination (MMSE), the Montreal cognitive assessment (MoCA), and the geriatric depression scale (GDS).

### 2.2. Clinical Instruments for Data Collection

MDS-UPDRS part III. This scale is used for the assessment of function in PD. UPDRS part III measures motor functions. It consists of 14 items with 27 questions, each scored from 0 to 4. Total scores for the UPDRS part III range from 0 to 108, with higher scores indicating greater motor symptoms/impairment [10].

MADRS. This scale is to evaluate MDD and includes nine items that the patient rates on a scale from 0 to 6: reported sadness, inner tension, reduced sleep, reduced appetite, concentration difficulties, lassitude, inability to feel, pessimistic thoughts, and suicidal thoughts. Higher scores indicate more severe MDD, and the maximum score is 54. MADRS is especially sensitive to changes and is, therefore, suitable for measuring the effect of treatments [11]. The scale has been validated in its Spanish language version, showing good psychometric properties, similar to those of the original scales [12].

MMSE. This is the most commonly used brief cognitive tool in the assessment of a variety of cognitive disorders. The tool comprises a short battery of 20 individual tests covering 11 domains with a maximum score of 30 points. Completion time is usually 8 min in cognitively unimpaired individuals and up to 15 min in patients with dementia. However, the main psychometric issue concerns MMSE’s diagnostic validity against dementia, mild cognitive impairment, and delirium. Internal consistency appears to be moderate, and the test–retest reliability is good [13]. This scale has been validated in the Mexican population [14].

MoCA. This test has been shown to be a highly effective tracking tool for discriminating between normal cognitive function and mild cognitive impairment and early onset dementia [15]. The average time taken to administer the test is ten to fifteen minutes. The main advantage of MoCA is its sensitivity in detecting mild cognitive impairment (MCI) and mild Alzheimer’s disease (90% and 100%, respectively) [15]. MoCA is a valid and reliable instrument for MCI and dementia-screening in the Mexican population, even after adjusting for age and education [16].

### 2.3. Statistical Analysis

The descriptive statistics, including the totals, proportions, and frequencies, were obtained from the categorical and ratio variables. In addition, central tendency and dispersion measures were obtained from the numerical variables. Statistical significance was evaluated using statistical hypothesis tests, by comparing proportions for nominal variables (chi-squared), and mean-comparison tests for numerical data (Student’s *t*-test). Subsequently, logistic regression modeling was performed when dependent variables were binary, and the odds ratio (OR) was calculated [17]. All the statistical analyses were conducted using the Stata 14^®^ program. Values with *p* < 0.05 were taken as statistically significant.

## 3. Results

### 3.1. Clinical and Sociodemographic Variables

Table 1 shows the clinical and sociodemographic variables in our study. As regards age, the mean was 58.49 ± 11.02 years and the mean age of onset of PD was 50.66 ± 11.86 years. This neurodegenerative disease was more frequent in men (57%, *n* = 57) than in women. Education beyond high school was reported in 35% of the patients (*n* = 35), 40% (*n* = 40) were economically productive, 68% (*n* = 68) were married, 30% (*n* = 30) had a history of psychiatric illness, 66% (*n* = 66) consumed caffeine, and 15% (*n* = 15) used tobacco. Regarding the years of evolution of PD, the mean was 7.83 ± 5.33 years, 13% (*n* = 13) had a family history of PD, and 67% (*n* = 67) had no comorbidities. Regarding diabetes type 2 (T2D) and arterial hypertension (AH), 11% (*n* = 11) had both T2D and AH. The most frequent symptom at the onset of the disease was tremors (67%, *n* = 67), and the most common side of onset was the right side (57%, *n* = 57). UPDRS part III, which measures motor functions in PD, resulted in a mean score of 33.67 ± 5.67. The mean score for MADRS was 21.33 ± 5.49. For cognitive assessment, the MoCA mean score was 21.06 ± 4.65, and MMSE was 26.67 ± 1.20. For the severity of MDD in the sample, using the MADRS score, 51% (*n* = 51) of the patients were classified as suffering from mild MDD, 44% (*n* = 44) with moderate MDD, and 5% (*n* = 5) with severe MDD.

### 3.2. Medication Variables

As seen in Table 2, selective serotonin reuptake inhibitors (SSRIs) were the most frequent antidepressant treatment (69%). To treat PD, levodopa/carbidopa (80%) and pramipexole (52%) were highly used. In our study, some subjects were under treatment with dual antidepressants, as well as more than one PD medication.

### 3.3. Comparison between Mild and Moderate–Severe MDD Groups

Table 3 shows the results of the comparisons between the variables of both groups. The variables that showed statistically significant differences were rigidity (12.77% vs. 36.17%, *p* = 0.005), gait disturbances (12.77% vs. 2.13%, *p* = 0.023), left side of onset (31.91% vs. 48.94%, *p* = 0.037), and MADRS score (13.43 ± 3.79 vs. 25.44 ± 5.66).

### 3.4. Comparison between Sexes in General Sample

Table 3 shows the results of the hypothesis tests between the variables of both groups (male–female). The clinical variables that showed statistically significant differences were the MADRS score (18.41 ± 7.29 vs. 21.31 ± 8.10, *p* = 0.028) and the MMSE score (26.22 ± 2.97 vs. 25.03 ± 3.58, *p* = 0.043).

The sociodemographic variables that showed statistically significant differences were education superior to high school (43.64% vs. 23.08% *p* = 0.043), married status (80.00% vs. 51.28%, *p* = 0.002), and tobacco use (23.64% vs. 2.56%, *p* = 0.003). When the drug variables were compared, no statistically significant differences were found between the groups.

### 3.5. Comparison between Onset with Tremor and Onset with Other Symptoms

Supplementary Materials Table S1 shows the results of the hypothesis tests between the variables of both groups (onset with tremor–onset with other symptom). The clinical variables that showed statistically significant differences were age (60.33 ± 11.04 vs. 54.39 ± 10.16, *p = 0.006*), age of onset (52.91 ± 12.10 vs. 45.65 ± 9.95, *p = 0.002*), and AH (14.49% vs. 3.23%, *p = 0.044*). No other variables showed statistically significant differences between groups.

### 3.6. Independent Logistic Regressions for Binary Dependent Variables

In the association analysis between M/S MDD and different variables, the only variable that was associated with moderate–severe MDD was rigidity at the onset of the disease (OR = 3.75, *p* = 0.013). This association persisted when the analysis was realized by sex and was done for the male group (OR = 4.39, *p* = 0.047).

## 4. Discussion

The current study reported the clinical and sociodemographic factors affecting MDD among patients with PD in the outpatient clinic of a national neurological referral center in Mexico.

Traditionally, MDD has been considered a predominantly female disease, with a two-fold greater prevalence than what is found in the male population. This observation is independent of country and culture [18]. In PD, depressive symptoms are reported in approximately 20% to 30% of the patients, and being female is a risk factor for presenting these symptoms [19]. In a large study that included more than 1400 patients, MDD was more common in the female than in the male patients and was more prevalent in individuals in the advanced stages of PD and those with dementia than in the patients with less severe disease [20]. In our study, we found a male predominance of PD with MDD (57%). However, most cases of MDD in male subjects were of a mild severity (56%) compared to female cases, which were moderate to severe (49% and 7%, respectively). This means that the severity of MDD in our subjects was greater in the female population compared to the male population, although, in comparing the groups, there were no statistically significant differences between them. In a study realized by Kahlil et al. (2018), similar findings were encountered. They found a male predominance of MDD in patients with PD (71.9%) [3].

Marital status has been addressed in various studies of PD-related MDD, without finding any association between this variable and the disease [3,21,22]. In our study, we encountered no association between the severity of the MDD and marital status. In addition, there was no difference between mild MDD and the moderate/severe groups when compared. However, when the male and female groups were compared, we encountered a statistically significant difference between the groups (*p* = 0.002), with the male group having a greater predominance of married status (80%) in comparison with the female group (51%). This variable should be addressed in future studies, especially in our population, since other factors, such as life expectancy and cultural beliefs, are different from those in other countries.

Another variable that has been studied and that contributes to the multifactorial nature of MDD in PD is educational level. The evidence encountered in some studies is controversial. In a study carried out by Eydivandi et al. (2021), an association was found between higher educational level and MDD (*p* < 0.05) [23]. On the other hand, Khalil et al. [3] reported no difference when comparing the educational levels of depressed and non-depressed groups (*p =* 0.134). In addition, no association was found between educational level and MDD in PD. In another study performed recently by Lian et al. (2019), when comparing the group without MDD with the depressed group in PD, the group with MDD showed a significantly lower education level [24]. In our study, no differences were found in educational level higher than high school when comparing the groups of mild MDD and moderate to severe MDD. On the other hand, when comparing the male and female groups, we found that the male group was more likely to have been educated beyond high school in comparison with the female group (43.64% vs. 23.08%, *p =* 0.043). This difference between the sexes could be attributable to cultural beliefs among the population, which limit access to adequate education for females.

Tremor corresponds to one of the cardinal symptoms of PD (stiffness, bradykinesia, and postural instability). Tremor is commonly the first symptom to appear in PD, being found in up to 90% of the patients throughout their lives [25]. In the present study, we found that 67% of the sample (*n* = 67) started PD with tremor. When we compared the groups of patients who started with tremor and those who started with other symptoms, we found that those who started with tremor were older at the time of the study and at the onset of the disease. Some studies, when comparing groups of tremor predominance vs. other motor symptoms, have not found statistically significant differences in terms of age and age of onset of the disease [26,27]. Similarly, when we made the comparison between onset with tremor (OWT) and onset with other symptoms (OWOS), we found that the group that begins only with tremor had a greater number of patients affected with AH (14.49%, *n* = 10). Some studies have tried to find some association between AH and the risk of developing PD; however, no association has been found between hypertension and PD [28,29]. These results could be attributed to the fact that the Mexican population is different from the populations of other studies, mainly in that this population has a high prevalence in AH, so this variable could behave as a risk factor in this particular population. Given the above, it would be interesting to address this variable as a possible risk factor in the Mexican population in subsequent studies.

Among the clinical variables that have been studied for their association with MDD and PD are rigidity and gait disturbances. In a study performed by Papapetropoulos et al. (2005), MDD was associated with severity of bradykinesia and axial rigidity [30]. In addition, another study carried out by Reijnders et al. (2009) showed that non-tremor-dominant PD, which is characterized by hypokinesia, rigidity, postural instability, and gait disorder, is associated with cognitive deterioration, MDD, apathy, and hallucinations [31]. In our study, when comparing the group of mild symptoms with the moderate to severe symptoms group, a statistically significant difference was found (12.77% vs. 36.17%, *p =* 0.005) in rigidity at the onset of disease. When realizing the logistic regressions for binary dependent variables, we found an association between rigidity at the onset of disease and moderate to severe MDD (OR = 3.75, *p =* 0.013). This association persisted in the analysis when adjusted for male sex (OR = 4.39, *p =* 0.047). There was no association with moderate to severe MDD in the females. Rigidity is an important symptom to assess because it can cause long-lasting psychological effects that could worsen MDD [32]. On the other hand, there are studies that have addressed gait disturbances. In a study carried out by Kincses et al. (2017), MDD in patients with PD was associated with gait components [33]. In our study, we only found differences between the groups of mild and moderate to severe depressed patients in gait disturbances at the onset of disease, with a predominance in the first group (12.77% vs. 2.13%, *p* = 0.023). These results may be associated with greater severity of rigidity in the late stages of PD, with a chronic evolution.

The side of onset of the disease has also been studied for its association with MDD in PD. In some studies, no differences were encountered between MDD in PD and side of onset of the disease (left, right, bilateral) when compared with patients with PD and no MDD [23,34]. In our sample, when comparing the group with mild MDD with the moderate to severe MDD group, a statistically significant difference was found between the groups for the left side of the onset variable (31.91% vs. 48.94, *p =* 0.037). This means that, in our sample patients with PD and severe to moderate MDD, the onset was predominantly on the left side.

Another variable to take into account is tobacco consumption. In our study, when the female group was compared to the male group, the consumption of tobacco was greater in the male group, with statistically significant differences (23.64% vs. 2.56%, *p* *=* 0.003). In a study realized by Khalil et al. (2018), no differences were found between males when comparing the group with MDD in PD with a non-depressed PD group (*p =* 0.415) [3].

As regards cognitive evaluation with MoCA and MMSE, it has been reported in the literature that patients with PD can show normal scores in MMSE while having MoCA scores compatible with MCI and cognitive impairment. In a study carried out by Vásquez et al. [35], 80% of the studied sample had MCI, with an average score in the MoCA test of 20.7. However, the average score of the sample using the MMSE test was 26.7, which means that there was no cognitive impairment represented in this score. The authors concluded that MoCA may be a good screening test for patients with PD who do not present cognitive complaints with a normal score on an MMSE test. In our study, the average score for the MoCA test was 21.33, and, for the MMSE, it was 26.67. This means that, according to the MoCA scores, the sample showed mild cognitive impairment, which contrasted with the sample’s average score using MMSE, which indicated no cognitive impairment. These findings are similar to those in the above-mentioned study and must be interpreted carefully because of the influence of age and level of education on the test scores, especially in the MMSE [36]. Additionally, our sample was diagnosed with co-morbid MDD, which can worsen the cognitive symptoms that accompany PD.

Selective serotonin reuptake inhibitors have traditionally been used for the treatment of MDD due to the adequate safety profile that these drugs provide. However, these drugs can worsen tremors in up to 5% of patients with PD [37]. On the other hand, dual antidepressants for the treatment of MDD in PD are considered “clinically useful” due to their superior effect compared to placebos in clinical trials [38]. In our study, 69% of the sample was under treatment with some selective serotonin reuptake inhibitor, and 20% were under treatment with dual antidepressants. From these results, we can see the tendency in our center is to treat MDD with selective serotonin reuptake inhibitors; however, a large portion of the patients were already starting to be treated with dual antidepressants. This last population will serve as the basis for future follow-up, response to treatment, and safety profile studies. In addition to dual antidepressants and selective serotonin reuptake inhibitors, the patients were also treated with tricyclic antidepressants and mirtazapine, as well as the respective antiparkinsonic treatment, the latter being highly variable between patients. When the drugs used for both MDD and PD were compared between the sexes (male–female) and MDD severity, no statistically significant difference was found.

This study has limitations that should be mentioned, such as the sample size. It is a study with a non-probabilistic sample. Similarly, various types of PD were included in the analysis, and the sample was obtained at a third level of attention, which limits the interpretation and generalization of the outcomes. Another limitation is that the comparison analyses were carried out between the sexes and severity of MDD and there was no comparison with a control group without MDD. Clinically, anxiety was not evaluated in this study, which is a limitation since it is a frequent comorbidity that could be exacerbating depressive symptoms. Considering the average age of onset (50.66 ± 11.86) and years of evolution (7.83 ± 5.33) of the sample, we must also take as a limitation what some authors have pointed out, that there could be an overlap between PD and progressive supranuclear palsy–-parkinsonism predominance (PSP-P) if only clinical criteria are considered. Due to the above, there is a possibility that some cases of PSP-P were considered as PD [39,40].

## 5. Conclusions

In this study, we aimed to describe the sociodemographic and clinical variables of PD patients diagnosed with MDD at the outpatient clinic of a national neurological referral center. In our results, we found that the males were more prone to MDD than the females, although the severity was found to be higher in the female Mexican population. Cognitive impairment was worse in the females. The M/S MDD prevalence was as high as 49% in the PD patients. Rigidity at the onset of the disease was the only clinical variable that was associated with M/S MDD. The differences found between the sexes, as well as between the groups by severity of MDD, can be attributed to study limitations, such as the sample size. Therefore, it would be advisable for future studies to take this into account. However, our findings are important because they can serve as a guideline for further analyses, as well as for clinicians to consider populations that may be at risk for developing MDD in the context of PD. This is aimed at improving the quality of life of these patients, as well as their long-term results in the evolution of the disease. We stress the importance of raising awareness regarding MDD in PD.

## Figures and Tables

**Table 1:** Sociodemographic variables of the general sample (*n* = 100).

Variables	Results
Age (years, average ± SD)	58.49 ± 11.02
Age at onset of PD (years, average ± SD)	50.66 ± 11.86
Years of PD evolution (average ± SD)	7.83 ± 5.33
UPDRS III (average ± SD)	33.67 ± 5.67
MADRS (average ± SD)	21.33 ± 5.49
MoCA (average ± SD)	21.06 ± 4.65
MMSE (average ± SD)	26.67 ± 1.20
Sex	
Male, % (*n*)	57 (57)
Female, % (*n*)	43 (43)
Diagnosis	
PD, % (*n*)	65 (65)
Early onset PD, % (*n*)	23 (23)
Youth PD, % (*n*)	3 (3)
Family PD, % (*n*)	6 (6)
Not defined, % (*n*)	2 (2)
Presence of family history	13 (13)
PPH	
None, % (*n*)	67 (67)
T2D, % (*n*)	11 (11)
AH, % (*n*)	11 (11)
Other, % (*n*)	18 (18)
Symptoms at the beginning of the disease	
Tremor, % (*n*)	67 (67)
Rigidity, % (*n*)	24 (24)
Gait disturbances, % (*n*)	7 (7)
Strength disturbances, % (*n*)	5 (5)
Bradykinesia, % (*n*)	2 (2)
Side of onset of the disease	
Right, % (*n*)	57 (57)
Left, % (*n*)	40 (40)
Bilateral, % (*n*)	2 (2)
Education higher than high school, % (*n*)	35 (35)
Economically productive, % (*n*)	40 (40)
Married, % (*n*)	68 868)
History of psychiatric illness, % (n)	30 (30)
Consumes caffeine, % (*n*)	66(66)
Consumes tobacco, % (*n*)	15 (15)
Severity of MDD by MADRS	
Mild, % (*n*)	51 (51)
Moderate, % (*n*)	44 (44)
Severe, % (*n*)	5 (5)
Cases of moderate to severe depression, % (*n*)	49 (49)

PD: Parkinson’s disease, UPDRS III: unified Parkinson´s disease rating scale, MADRS: Montgomery–Asberg depression rating scale, MoCA: Montreal cognitive assessment, MMSE: mini mental state examination, PPH: personal pathologic history, T2D: type 2 diabetes, AH: arterial hypertension, *n*: number of patients, SD: standard deviation. Some items do not add up to 100% because they could have more than one of the conditions.

**Table 2:** Drug variables in the general sample (*n* = 100).

Variable	% (*n*)
Antidepressant management	
SSRIs	69 (69)
Dual antidepressant	20 (20)
Mirtazpine	5 (5)
Tricyclic antidepressant	13 (13)
Trazodone	1 (1)
PD management	
Donepezil	1 (1)
Pramipexole	52 (52)
Galantamine	1 (1)
Bromocriptine	4 (4)
Trihexiphenidyl	2 (2)
Leflunomide	1(1)
Rotigotine	5 (5)
Levodopa	2 (2)
Levodopa/Carbidopa	80 (80)
Levodopa/Benserazide	5 (5)
Levodopa/Carbidopa/Entacapona	13 (13)
Selegiline	7 (7)
Rasagiline	1 (1)
Amantadine	22 (22)
Biperiden	13 (13)
Propanolol	3 (3)

SSRIs: selective serotonin reuptake inhibitors, PD: Parkinson’s disease, *n*: number of patients. Some items do not add up to 100% because they could have more than one of the conditions.

**Table 3:** Comparison between sexes and severity of MDD in general sample (*n* = 100) of patients with PD and MDD (hypothesis tests).

Variable	Male (*n* = 57)	Female (*n* = 43)	*p* < 0.05	Mild MDD (*n* = 51)	M/S MDD (*n* = 49)	*p* < 0.05
Age (years, average ± SD)	58.82 ± 11.42	58.05 ± 10.71	0.365	58.57 ± 10.75	57.92 ± 11.47	0.387
Age of onset PD (years, average ± SD)	50.65 ± 12.20	50.67 ± 11.68	0.496	50.96 ± 11.41	49.6 ± 12.47	0.291
Years of evolution PD (average ± SD)	8.18 ± 4.84	7.37 ± 6.00	0.230	7.62 ± 5.00	8.31 ± 5.84	0.268
UPDRS III (average ± SD)	29.80 ± 16.37	44.00 ± 16.03	0.188	28.35 ± 14.22	33.73 ± 18.1	0.134
MADRS (average ± SD)	18.41 ± 7.29	21.31 ± 8.10	**0.028**	13.43 ± 3.79	25.44 ± 5.66	**<0.001**
MOCA (average ± SD)	21.52 ± 4.51	20.41 ± 4.91	0.122	21.4 ± 4.51	20.77 ± 4.94	0.258
MMSE (average ± SD)	26.22 ± 2.97	25.03 ± 3.58	**0.043**	25.93 ± 2.92	25.51 ± 3.63	0.281
Sex						
Male	-	-	NA	62.75 (32)	51.06 (25)	0.085
Female	-	-	NA	37.25 (19)	48.94 (24)	0.085
PPH						
Presence of family history, % (*n*)	12.73 (7)	12.82 (6)	0.403	10.64 (5)	14.89 (7)	0.281
None, % (*n*)	69.09 (39)	64.10 (28)	0.288	61.70 (31)	72.34 (35)	0.202
T2D, % (*n*)	14.55(8)	5.13 (2)	0.134	10.64 (5)	10.64 (5)	0.347
AH, % (*n*)	7.27 (4)	15.38 (7)	0.070	8.51 (4)	12.77 (6)	0.395
Other, % (*n*)	14.55(8)	23.08 (10)	0.178	23.40 (12)	12.77 (6)	0.125
Symptoms at the onset of the disease						
Tremor, % (*n)*	36 (63.64)	71.79 (31)	0.154	74.47 (37)	59.57 (29)	0.072
Rigidity, % (*n*)	11 (20.00)	30.77 (13)	0.103	12.77 (6)	36.17 (18)	**0.005**
Gait disturbances, % (*n*)	7.27 (4)	7.69 (3)	0.497	12.77 (6)	2.13 (1)	**0.023**
Strength disturbances, % (*n*)	7.27 (4)	2.56 (1)	0.143	4.26 (2)	6.38 (3)	0.332
Bradykinesia, % (*n*)	1.82 (1)	2.56 (1)	0.420	2.13 (1)	2.13 (1)	0.494
Side of onset of the disease						
Right, % (*n*)	54.55 (31)	61.54 (26)	0.292	63.83 (32)	51.06 (25)	0.087
Left, % (*n*)	41.82 (24)	38.46 (17)	0.421	31.91 (16)	48.94 (24)	**0.037**
Bilateral, % (*n*)	3.64 (2)	0.00 (0)	0.107	4.26 (2)	0.00 (0)	0.074
Sociodemographic variables						
Education higher than high school, % (*n*)	43.64 (25)	23.08 (10)	**0.043**	29.79 (15)	40.43 (20)	0.158
Economically productive, % (*n*)	38.18 (22)	43.59 (19)	0.165	46.81 (23)	34.04 (17)	0.09
Married, % (*n*)	80.00 (46)	51.28 (22)	**0.002**	68.09 (34)	68.09 (33)	0.472
History of psychiatric illness, % (*n*)	23.64 (13)	38.46 (17)	0.086	23.40 (12)	36.17 (18)	0.1
Consumes caffeine, % (*n*)	69.09 (39)	61.54 (26)	0.250	65.96 (33)	65.96 (32)	0.471
Consumes tobacco, % (*n*)	23.64 (13)	2.56 (1)	**0.003**	21.28 (11)	8.51 (4)	0.073
Severity of MDD by MADRS						
Mild, % (*n*)	56.14 (32)	44.19 (19)	0.194	-	-	NA
Moderate, % (*n*)	40.35 (23)	48.84 (21)	0.163	-	-	NA
Severe, % (*n*)	3.51 (2)	6.98 (3)	0.188	-	-	NA
Drug variables						
SSRI, % (*n*)	70.18 (40)	72.09 (31)	0.417	74.51 (38)	67.35 (33)	0.215
Dual, % (*n*)	17.54 (10)	20.93 (9)	0.335	13.73 (7)	24.49 (12)	0.085
Mirtazapine, % (*n*)	8.77 (5)	2.33 (1)	0.090	9.80 (5)	2.04 (1)	0.051
Tricyclic, % (*n*)	10.53 (6)	13.95 (6)	0.301	11.76 (6)	12.24 (6)	0.471
Trazodone, % (*n*)	0.00 (0)	2.33 (1)	0.124	1.96 (1)	0.00 (0)	0.162
Donepezil, % (*n*)	0.00 (0)	2.33 (1)	0.124	1.96 (1)	0.00 (0)	0.162
Pramipexole, % (*n*)	47.37 (27)	53.49 (23)	0.272	45.10 (23)	55.10 (27)	0.159
Galantamine, % (*n*)	0.00 (0)	2.33 (1)	0.191	0.00 (0)	2.04 (1)	0.153
Bromocriptine, % (*n*)	3.51 (2)	4.65 (2)	0.386	5.88 (3)	2.04 (1)	0.164
Trihexiphenidyl, % (*n*)	1.75 (1)	2.33 (1)	0.580	0.00 (0)	4.08 (2)	0.073
Leflunomide, % (*n*)	0.00 (0)	2.33 (1)	0.124	1.96 (1)	0.00 (0)	1.000
Rotigotine, % (*n*)	7.02 (4)	6.98 (3)	0.503	5.88 (3)	8.16 (4)	0.328
Levodopa, % (*n*)	3.51 (2)	0.00 (0)	0.215	1.96 (1)	2.04 (1)	0.489
Levodopa/Carbidopa, % (*n*)	80.70 (46)	79.07 (34)	0.420	84.31 (43)	75.51 (37)	0.136
Levodopa/Benserazide, % (*n*)	5.26 (3)	74.42 (32)	0.445	3.92 (2)	67.35 (33)	0.307
Levodopa/Carbidopa/Entacapona, % (*n*)	12.28 (7)	13.95 (6)	0.403	13.73 (7)	12.24 (6)	0.413
Selegiline, % (*n*)	3.51 (2)	13.95 (6)	0.057	5.88 (3)	10.20 (5)	0.213
Rasagiline, % (*n*)	3.51 (2)	0.00 (0)	0.107	1.96 (1)	2.04 (1)	0.489
Amantadine, % (*n*)	19.30 (11)	25.58 (11)	0.226	23.53 (12)	20.41 (10)	0.353
Biperiden, % (*n*)	15.79 (9)	9.30 (4)	0.170	13.73 (7)	12.24 (6)	0.413
Propanolol, % (*n*)	3.51 (2)	2.33 (1)	0.366	1.96 (1)	4.08 (2)	0.267

MDD: major depressive disorder, M/S: moderate to severe, PD: Parkinson’s disease, UPDRS III: unified Parkinson´s disease rating scale, MADRS: Montgomery–Asberg depression rating scale, MoCA: Montreal cognitive assessment, MSSE: mini mental state examination, PPH: personal pathologic history, T2D: type 2 diabetes, AH: arterial hypertension, SSRI: selective serotonin reuptake inhibitor, SD: standard deviation. Some items do not add up to 100% because they could have more than one of the conditions. Statistically significant results are shown in bold and cursive letters.

## Data Availability

The data supporting the current study are available from the corresponding author upon reasonable request.

## Supplementary Materials

The following are available online at https://www.mdpi.com/article/10.3390/brainsci12030326/s1, Table S1: Comparison between patients who started with tremor and those who started with another symptom (Hypothesis test).
