# Supplementary material for: Naturalistic Study of Depression Associated with Parkinson’s Disease in a National Public Neurological Referral Center in Mexico
Source: Brain Sci. 2022 Feb 28;12(3):326. doi: 10.3390/brainsci12030326 (PMC8945969; doi:10.3390/brainsci12030326)
Supplement: Supplementary file 1 [file brainsci-12-00326-s001.zip › brainsci-1595117-supplementary.pdf]

Supplementary Materials

Table S1. Comparison between patients who started with tremor and those who started with another symptom (Hypothesis test).

Variable	*OWT (n = 69)*	*OWOS (n = 31)*	*p* < 0.05
**Age**	60.33 ± 11.04	54.39 ± 10.16	***0.006***
**Age of onset PD**	52.91 ± 12.10	45.65 ± 9.95	***0.002***
**Yers of evolution PD**	7.42 ± 5.14	8.74 ± 5.79	*0.128*
UPDRS III	32.05 ± 15.43	30.83 ± 17.87	*0.402*
MADRS	18.81 ± 7.53	20.90 ± 7.91	*0.109*
**MOCA**	20.92 ± 4.64	21.35 ± 4.80	*0.337*
**MMSE**	25.75 ± 3.38	25.64 ± 2.98	*0.444*
**PPH**			
Presence of family history	11.59 (8)	16.13 (5)	*0.266*
None	62.32 (43)	74.19 (23)	*0.163*
T2D	10.14 (7)	12.90 (4)	*0.360*
AH	14.49 (10)	3.23 (1)	***0.044***
Other	18.84 (13)	12.9 (4)	*0.215*
**Side of onset of the disease**			
Right	53.62 (37)	58.06 (18)	*0.340*
Left	44.93 (31)	38.71 (12)	*0.281*
Bilateral	1.45 (1)	3.23 (1)	*0.279*
**Sociodemographic variables**			
Education higher than highschool	31.88 (22)	41.94 (13)	*0.165*
Married	63.77 (44)	74.19 (23)	*0.153*
Economically productive	44.93 (31)	32.26 (10)	*0.117*
Consumes caffeine	63.77 (44)	67.74 (21)	*0.384*
Cosumes tobacco	18.84 (13)	12.90 (4)	*0.224*
**Severity of depression by MADRS**			
Mild	56.52 (39)	38.71 (12)	***0.050***
Moderate	39.13 (27)	54.84 (17)	*0.072*
Severe	4.35 (3)	6.45 (2)	*0.328*

OWT: Onset with Tremor, OWOS: Onset with Other Symptom, MDD: Major Depressive Disorder, M/S: Moderate to Severe, PD: Parkinson’s Disease, UPDRS III: Unified Parkinson´s Disease Rating Scale, MADRS: Montgomery Asberg Depression Rating Scale, MoCA: Montreal Cognitive Assessment, MMSE: Minimental State Examination, PPH: Personal Pathologic History, T2D: Type 2 Diabetes, AH: Arterial Hypertension, SSRI: Selective Serotonin Reuptake inhibitor, SD: Standard Deviation. *Some items do not add up to 100% because they could have more than one of the conditions.* Statistically significant results are shown in bold and cursive letters.
